# Knitted Dish Cloths

**Published:** 1889-05

**Authors:** 


### KNITTED DISH-CLOTHS.

These are very nice to use, and cleaner than an ordinary dish-cloth, as they wash out more easily. Buy some very soft unbleached knitting cotton, and a pair of steel knitting needles, No. 8. Cast on fifty stitches. and knit backwards and forwards until you have done fifty rows, or your piece of knitting is square. Now take a bone crochet needle, and work in double crochet all around the knitted square, making five chain and then double crochet again at one corner to form a loop by which to hang the cloth up. Fasten off securely, and it is done. A packet of half a dozen of these cloths is sure to be welcome to anyone who has a house of her own. Another useful present which either boys or girls may make is a net formed of twine for keeping oranges or lemons hung up in. Any mother would be glad of a few of these for her store-room ; they are useful for so many things. Such presents as these cost very little money, and often give far more pleasure than those on which a lot of money has been spent.

